# Ipilimumab in Pretreated Patients With Advanced Malignant Melanoma: Results of the South African Expanded-Access Program

**DOI:** 10.1200/JGO.2016.006544

**Published:** 2016-11-02

**Authors:** Bernardo L. Rapoport, Daniel A. Vorobiof, Lydia M. Dreosti, Adam Nosworthy, Georgina McAdam, Johan P. Jordaan, Helen Miller-Jansön, Margreet de Necker, Janetta C. de Beer, Hennie Duvenhage

**Affiliations:** **Bernardo L. Rapoport**, Medical Oncology Centre of Rosebank; **Daniel A. Vorobiof**, Sandton Oncology Center; **Adam Nosworthy**, Wits Oncology Donald Gordon Medical Center; **Hennie Duvenhage**, Bristol-Myers Squibb South Africa, Johannesburg; **Lydia M. Dreosti**, University of Pretoria, Pretoria; **Georgina McAdam**, Rondebosch Oncology Medical Center, Cape Town; **Johan P. Jordaan**, Westridge Oncology Center, Durban; and **Helen Miller-Jansön**, **Margreet de Necker**, and **Janetta C. de Beer**, HEXOR, Midrand, South Africa.

## Abstract

**Purpose:**

The primary objective of this study was to evaluate 1- and 2-year survival rates and durable remissions in pretreated patients with advanced (unresectable or metastatic) malignant melanoma treated with ipilimumab in a South African expanded-access program (SA-EAP).

**Patients and Methods:**

This multicenter, retrospective study obtained data from pretreated patients with advanced malignant melanoma who were eligible for the ipilimumab SA-EAP. Ipilimumab was administered at a dose of 3 mg/kg intravenously every 3 weeks for four cycles to adults with advanced melanoma for whom at least one line of treatment for metastatic disease had failed. Data from the medical records of 108 patients treated within the SA-EAP were collected and statistically analyzed to determine overall (OS) and progression-free survival (PFS) at 1 and 2 years.

**Results:**

In the population of 108 patients, a median OS of 8.98 months (95% CI, 7.47 to 10.79 months) was observed. One-year OS was 36% (95% CI, 26% to 45%), and 2-year survival was observed as 20% (95% CI, 12% to 27%). The median survival without progression (ie, PFS) was 3.44 months (95% CI, 2.98 to 4.16 months), and 1- and 2-year PFS were 22% (95% CI, 14% to 29%) and 14% (95% CI, 8% to 21%), respectively. The longest recorded survival was 3.4 years. No independent prognostic variables were identified to predict for OS by multivariate Cox proportional hazards model.

**Conclusion:**

In this multicenter South African setting, ipilimumab at a dose of 3 mg/kg was an effective treatment with long-term OS in a subset of patients with pretreated advanced malignant melanoma.

## INTRODUCTION

In South Africa, melanoma is the eighth most common documented cancer in men, with a lifetime risk of one in every 186 men; in women, melanoma is the sixth most common cancer, with a lifetime risk of one in every 297 women. According to the South African National Cancer Registry (2005), whites are at higher risk; melanoma is the fifth most common cancer among white men and remains the sixth most common cancer in white women.^1^ Although evidence indicates that mortality rates are declining or stabilizing for certain populations or countries, partially because of heightened awareness and detection of disease at an earlier stage, mortality for advanced (unresectable or metastatic) melanoma remains high.^2-5^ Survival for stage IV disease, in particular, remains poor, with median survival times across studies ranging from 6 to approximately 12 months.^6,7^ Treatment of metastatic melanoma continues to represent a considerable unmet medical need on the basis of the rising worldwide incidence of the disease and the unsatisfactory efficacy and significant toxicities of currently available drugs.

Ipilimumab (YERVOY; Bristol-Myers Squibb, New York, NY) is a fully humanized monoclonal antibody directed against cytotoxic T-cell lymphocyte antigen-4 and the first treatment to demonstrate a survival benefit in advanced malignant melanoma. Efficacy data are largely based on results from two phase III clinical trials.^8,9^ The MDX010-20 study compared ipilimumab 3 mg/kg with an interventional vaccine, with increased median overall survival (OS) in the ipilimumab arm of 3.7 months (hazard ratio, 0.66; *P* = .003).^8^ The other study compared ipilimumab 10 mg/kg plus dacarbazine with dacarbazine alone, with higher 1-year OS in the ipilimumab arm (47.3% *v* 36.3%).^9^ A meta-analysis of 15 trials reported a median OS of 18.8 months with ipilimumab monotherapy (at 3 mg/kg) compared with 12.3 months with single-agent chemotherapy.^10^ Because of the limited data reported on the use of ipilimumab in metastatic malignant melanoma in developing countries, our retrospective study was undertaken to evaluate the long-term outcomes of ipilimumab, administered within a South African expanded access program (SA-EAP) in pretreated patients with this disease.

## PATIENTS AND METHODS

### Patient Population

Patients were enrolled in the SA-EAP according to the following criteria: histologically confirmed stage III (unresectable) or stage IV (metastatic) cutaneous, ocular or mucosal melanoma, or asymptomatic brain metastases resulting from melanoma; failure of or intolerance to at least one prior systemic treatment; age ≥ 18 years; and Eastern Cooperative Oncology Group performance status of 2 or less. Patients were excluded from the study on the basis of contraindication to ipilimumab therapy (eg, known autoimmune disease, HIV, hepatitis B or C); presence of symptomatic brain metastases; receipt of other concurrent systemic anticancer treatments for melanoma; or presence of another active concurrent malignant disease, with the exception of adequately treated basal or squamous cell skin cancer, superficial bladder cancer, or carcinoma in situ of the cervix.

### Study Design

In this multicenter retrospective study, data were extracted from patients’ medical records to evaluate the outcomes of ipilimumab treatment. Institutional ethics approval was obtained from the Human Sciences Research Council of South Africa.

### Data Collection and Statistical Analysis

The participating practices and academic institutions collected and recorded the required data on patient case report forms, which were returned to the study facilitator. The collected data were electronically captured on an Excel-based capturing tool (Microsoft, Redmond, WA) for analysis and analyzed using RStudio software (version 3.2.3; RStudio, Boston, MA).

Data from different points in time throughout a patient’s medical history were reviewed. These data included four aspects of treatment history: demographic features, disease characteristics, initial treatment at the time of enrollment in the SA-EAP, and courses of treatment. The data reported on patient- and disease-related factors, including: demographic information, melanoma subtype and stage, presence of brain or liver metastases, lactate dehydrogenase values, *BRAF* mutational status, ECOG performance status, and previous treatments.

Treatment-related information included history of concomitant drug use, details of ipilimumab treatment (date of first ipilimumab dose, number of infusions, date of reinduction, and reason for discontinuation or omission), date of first measured disease progression (PD), and overall tumor response rate after completion of induction therapy. Distribution of overall tumor response (proportion of patients with complete response [CR], partial response [PR], stable disease [SD], or PD) was defined according to WHO and RECIST at 12 and 24 weeks and every 3 months thereafter for up to 60 weeks.^11^ Disease control rate (DCR) was defined as the sum of the numbers of patients with PR, CR, and SD. Objective response rate was defined as the sum of the numbers of patients with CR and PR. Relevant biologic values and incidence and grading of adverse events (AEs) were also collected. A total of 247 patients from 35 participating centers were registered in the ipilimumab SA-EAP. Ten patients underwent treatment reinduction, and one patient was reinduced twice. CRFs were received for 108 patients from 21 centers.

The collected data were statistically analyzed using descriptive statistics, with medians and ranges of continuous variables and frequencies and percentages for categorical variables. OS and progression-free survival (PFS) were estimated using the Kaplan-Meier method, with 95% CIs reported.^12^ A Cox proportional hazards model was used to identify covariates independently associated with survival.

OS was analyzed using time from ipilimumab initiation date and date of most recent visit or death, whichever occurred first. PFS was analyzed using time from ipilimumab initiation date to end of follow-up or date of first measured PD or death resulting from any cause, whichever occurred first. Patients with neither PD nor death were right censored at the last date of tumor assessment. Time to progression was defined as time from ipilimumab initiation date to end of follow-up or date of first measured PD or treatment failure resulting from a competing risk (ie, death). Patients without PD (or death as competing risk) were right censored at the last date of tumor assessment.

## RESULTS

### Patients and Treatment

Patient characteristics at baseline are listed in Table 1. Of the 108 patients, 84 (78%) had cutaneous melanoma and 24 (22%) had noncutaneous melanoma, including uveal and mucosal melanomas and melanomas of unknown primary. All patients had received prior systemic therapy for advanced disease (median, one; range, one to five).

**Table 1:**
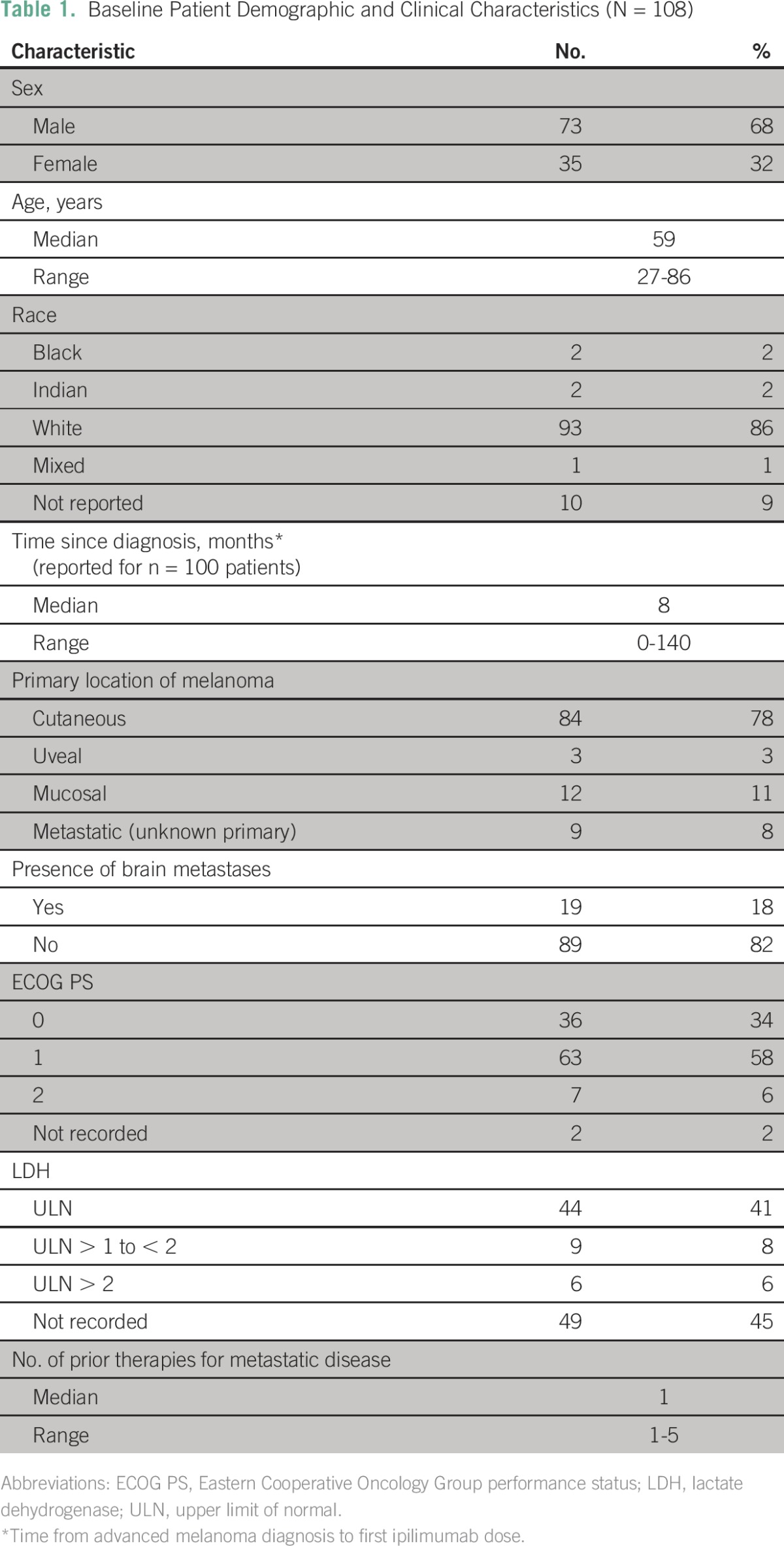
Baseline Patient Demographic and Clinical Characteristics (N = 108)

Regarding exposure to ipilimumab treatment and reinduction treatment, the median number of overall doses of ipilimumab treatment (n = 118) was four (range, one to four); the median length of ipilimumab treatment (n = 96) was 9 weeks (95% CI, 8 to 10). Ten patients were eligible for reinduction treatment; the median number of overall doses of ipilimumab reinduction treatment (n = 10) was four (range, one to four), and the median length of ipilimumab reinduction treatment (n = 8), defined as time from first ipilimumab reinduction treatment date to last ipilimumab reinduction treatment date, was 9 weeks (95% CI, 8 to 11 weeks).

Treatment discontinuation data were received for 46 patients (43%) who discontinued ipilimumab treatment permanently before having completed four doses of the drug. Two patients (2%) omitted a dose of ipilimumab. The main reason cited for discontinuation was death (48%), followed by PD (33%). One patient discontinued treatment because of severe immune GI toxicity. The median time to discontinuation of ipilimumab was 6 weeks.

### Efficacy

**Fig 1:**
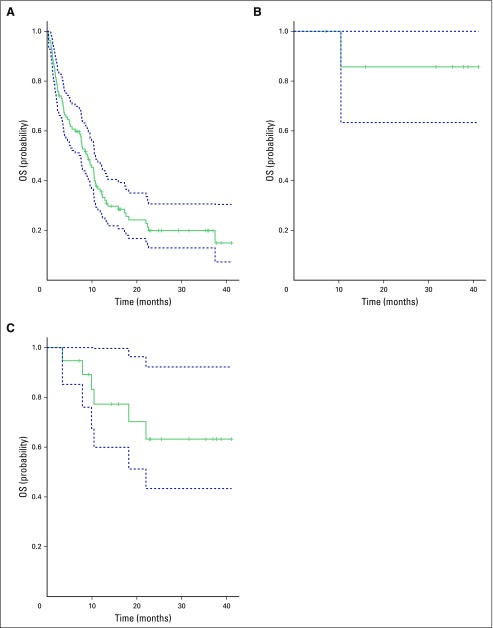
Kaplan-Meier plot of overall survival (OS) in the South African expanded-access program in (A) all patients, (B) patients who achieved complete response, (C) and patients without disease progression. Dashed lines indicate 95% CIs.

The median OS (n = 108) was 8.98 months (95% CI, 7.47 to 10.79 months), and the 1-year survival rate was 36% (95% CI, 26% to 45%). The 2-year survival rate was 20% (95% CI, 12% to 27%). The median PFS (n = 108) was 3.44 months (95% CI, 2.98 to 4.16 months). The 1-year PFS rate was 22% (95% CI, 14% to 29%). The 2-year PFS rate was 14% (95% CI, 8% to 21%). At a median follow-up of 24 months, the median survival of patients who attained a CR, PR, or SD was not reached (Figs 1A to 1C).

Cox multivariate analysis for OS and PFS of various patient characteristics, using stepwise backward elimination, failed to demonstrate any significant variable. The relationships between absolute lymphocyte count and absolute eosinophil count and both OS and PFS were also examined. Although there was a trend toward increased OS and PFS with high absolute lymphocyte and eosinophil counts, significance was not demonstrated (*P* > .05).

The median time to progression (n = 108) was 3.44 months (95% CI, 2.98 to 4.16 months). Seventy-three percent of patients had experienced PD by 10 months (95% CI, 65% to 82%), and 90% (95% CI, 84% to 96%) had experienced PD by 40 months. Analysis of best overall response rates (n = 62) showed that the DCR was 53% (95% CI, 41% to 66%). Best overall response rates are listed in Table 2. According to the timeframes and retrospectively captured data, the median time to best response for patients whose best response was PR was 12 weeks; for patients achieving a CR, it was 24 weeks. Individual best overall response rates for patients with mucosal melanoma, uveal melanoma, or metastatic melanoma of unknown primary site are listed in Table 3.

**Table 2:**
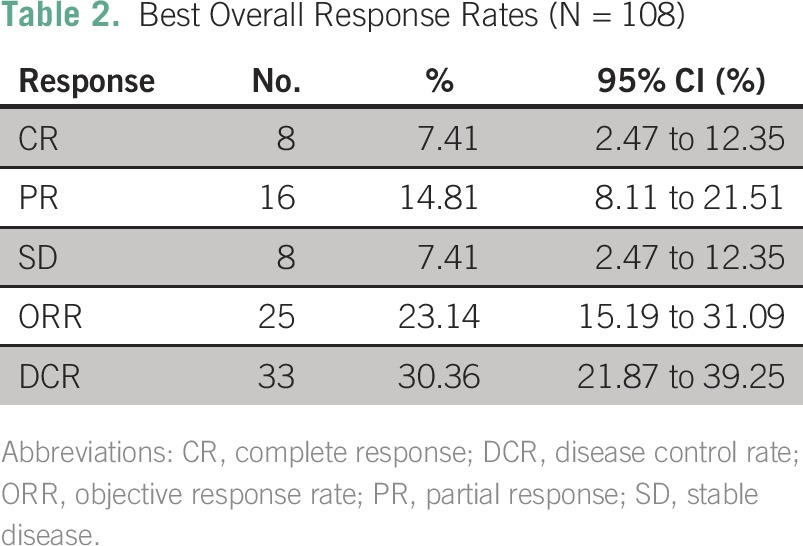
Best Overall Response Rates (N = 108)

**Table 3:**
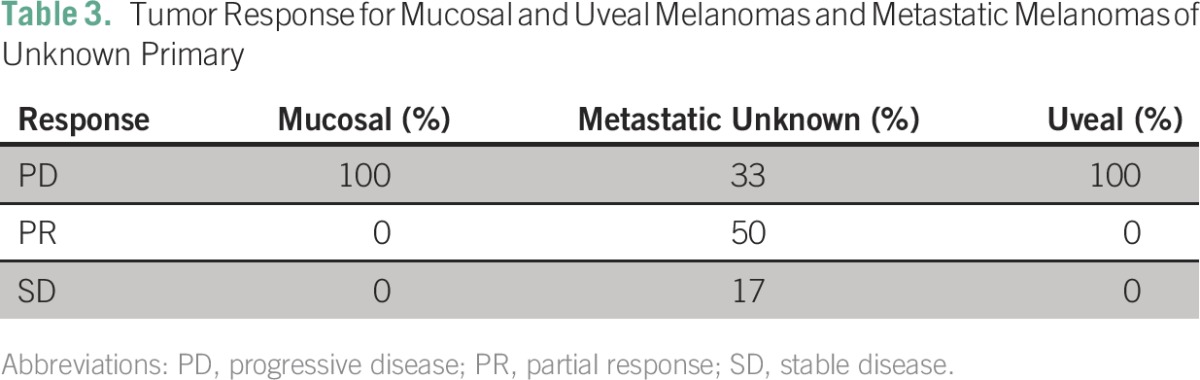
Tumor Response for Mucosal and Uveal Melanomas and Metastatic Melanomas of Unknown Primary

### AEs

Eleven severe AEs (grades 3 and 4) were reported; a majority of these were GI events. There were no reported deaths resulting from treatment-induced toxicities. Because these data were collected retrospectively, it is possible that toxicities were under-reported, particularly for grades 1 and 2. Additionally, and in view of the nature of the study, information on date of resolution and potential sequelae was not captured. AEs are summarized in Table 4.

**Table 4:**
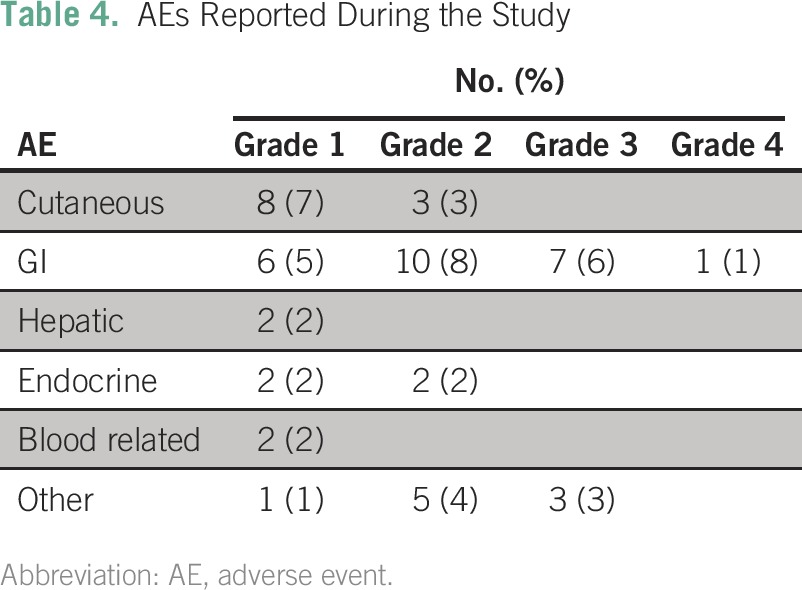
AEs Reported During the Study

## DISCUSSION

This retrospective analysis of the SA-EAP in a population of 108 patients with advanced malignant melanoma showed that patients treated with ipilimumab obtained a median OS of 8.98 months (95% CI, 7.47 to 10.79 months) and 1-year OS of 36% (95% CI, 26% to 45%). These results are comparable to the outcomes reported in the prospective phase III pivotal trial of ipilimumab at a dose of 3 mg/kg in pretreated patients with melanoma.^8^ It must be emphasized that before registration of ipilimumab, there was no evidence-based second-line treatment approved or recommended by South African guidelines for these patients.

These outcomes exceed suggested benchmark survival targets (proposed from a meta-analysis of second-line melanoma treatments) of 6.5 months for median OS and 25% for 1-year OS.^13^ The real-world survival data from our study are similar to those reported in an ipilimumab access scheme using a 10-mg/kg dosing regimen (median OS, 7.2 to 9 months).^13,14^ The median PFS of 3.44 months (95% CI, 2.98 to 4.16 months) was also similar to those reported in an Australian study of a similar nature (3.0 months; 95% CI, 2.7 to 3.4 months) and in another published access scheme study (95% CI, 2.6 to 4.3 months).

Overall, our results are in line with those previously reported for EAPs with ipilimumab at doses of 3 and 10 mg/kg. Those results are summarized in Table 5.^14-21^

**Table 5:**
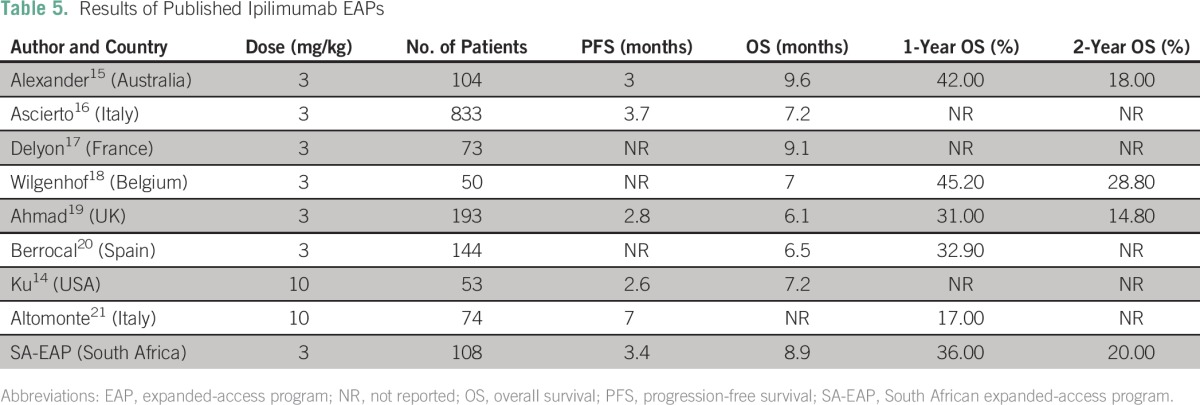
Results of Published Ipilimumab EAPs

One of the main weaknesses of our study relates to the retrospective nature of the investigation, the difficulties in controlling for selection bias, and the lack of a control group. A proportion of our patients did not complete ipilimumab treatment because of PD. Some of these patients may have had a short, predictable life expectancy with advanced disease. These patients are typically heavily overtreated and have limited therapeutic options and a lack of alternative options; they had an opportunity to receive ipilimumab within the framework of this EAP. As a result of the retrospective nature of the study, investigations of patients did not occur at predefined times; therefore, this may have resulted in over-reporting of DCR.

Some patients with melanoma treated with ipilimumab exhibit an initially increased size of tumor lesions, proved by biopsy as inflammatory cell infiltrates or necrosis, followed by a decreased tumor burden. These immune-related response patterns have been recognized in clinical trials of ipilimumab, including the documentation of new tumors associated with edema and infiltrates of immune cells and transient increases in baseline tumor lesions. Clinicians should be aware of the possible presence of pseudotumor progression, which could be regarded as PD.^22^ However, this effect occurs in less than 10% of patients and could not account for the early PD documented among the pretreated patients with metastatic melanoma accrued into our EAP.

Of the 108 patients, 43% discontinued ipilimumab permanently before having completed four doses. PD was cited as the most common reason for discontinuation of treatment. These results are comparable to those of other studies using a 3-mg/kg dose of ipilimumab (33% *v* 40.3%).^15-20^ Only 2% of patients discontinued treatment with ipilimumab because of severe immune-related AEs. This incidence is somewhat lower than percentages reported in the other studies (9.9% and 11.1%)^15-20^; however, the retrospective nature of our study might account for this observation.

Treatment with ipilimumab is associated with immune-related AEs. These immune-related AEs are related to the drug mechanism of action. In comparison with other published studies of ipilimumab, the overall incidence of grade 3 and 4 immune-related AEs (9.2%) in the SA-EAP was similar to that reported in the multicenter phase III randomized controlled trial conducted by Hodi et al.^8^ These data suggest that the toxicity profile in the real-world setting is not different from that observed in clinical trial settings. Additionally, the use of ipilimumab at a dose of 3 mg/kg has a better safety profile than at 10 mg/kg, with similar outcomes. Some patients discontinued treatment early, decreasing the chance of developing immune-related AEs, which may account for the lower incidence of immune-related AEs.

Prior reports have indicated that an increase in absolute lymphocyte count and absolute eosinophil count occurring 3 weeks after treatment initiation may be associated with improved survival. Our data failed to show that. However, this once again may be a result of the retrospective nature of our study. Future studies should address this issue to further characterize this observation. Prospective studies examining lymphocyte subpopulations are required.

**Fig 2:**
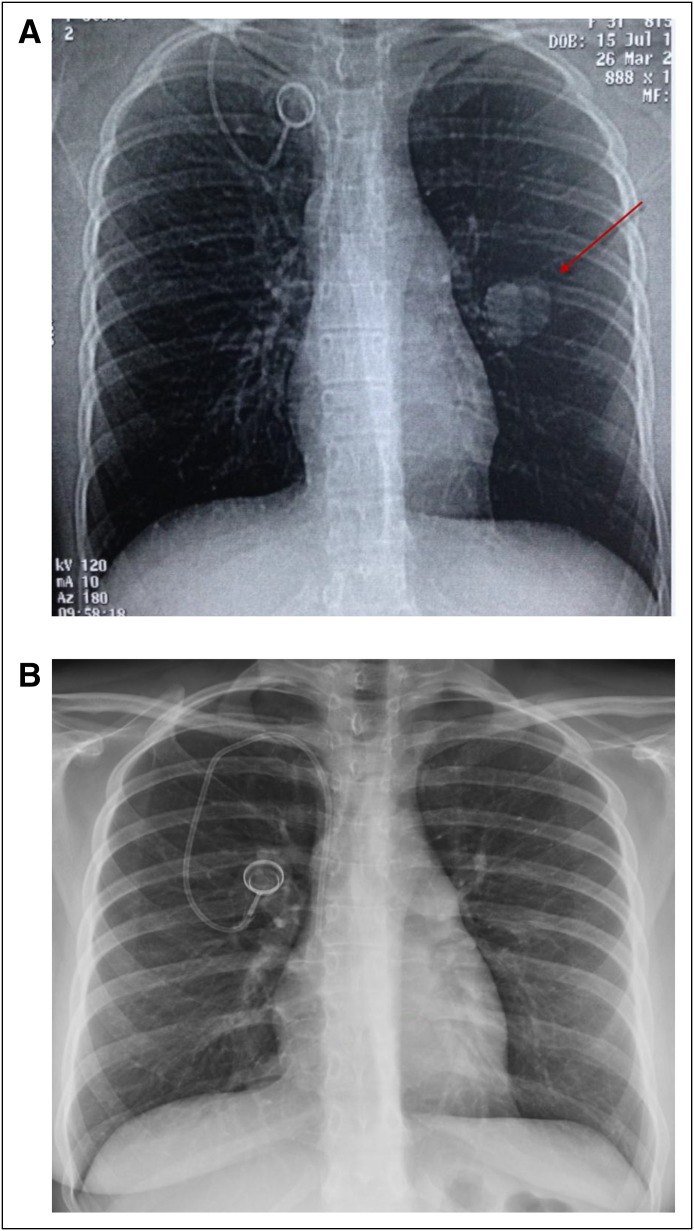
(A) Pre- (March 26, 2012; red arrow indicates tumor) and (B) post-treatment (July 17, 2013) chest x-rays. Patient remains in remission.

One of the most important aspects of treatment with checkpoint inhibitors is the improvement in survival and documentation of long-term durable remissions in some patients (Figs 2A and 2B). Future research should focus on improving the number of patients who will experience durable remissions and should include combination with other checkpoint inhibitors, gene therapy, vaccine therapy, and other immunostimulatory approaches.

In conclusion, our study of ipilimumab in the South African setting found that the efficacy and tolerability of ipilimumab at 3 mg/kg for the treatment of unresectable metastatic melanoma in pretreated patients align with data reported in several published studies with similar treatment doses and patient populations.
